# BRWD3 promotes KDM5 degradation to maintain H3K4 methylation levels

**DOI:** 10.1073/pnas.2305092120

**Published:** 2023-09-18

**Authors:** Dongsheng Han, Samantha H. Schaffner, Jonathan P. Davies, Mary Lauren Benton, Lars Plate, Jared T. Nordman

**Affiliations:** ^a^Department of Biological Sciences, Vanderbilt University, Nashville, TN 37212; ^b^Department of Computer Science, Baylor University, Waco, TX 76798; ^c^Department of Chemistry, Vanderbilt University, Nashville, TN 37212

**Keywords:** chromatin, Drosophila, methylation, ubiquitin, epigenetics

## Abstract

Histone modifications are critical for regulating chromatin structure and gene expression. Dysregulation of histone modifications likely contributes to disease states and cancer. Depletion of the chromatin-binding protein BRWD3 (Bromodomain and WD repeat-containing protein 3), a known substrate-specificity factor of the Cul4-DDB1 E3 ubiquitin ligase complex, results in increased H3K4me1 (H3 lysine 4 monomethylation) levels. The underlying mechanism linking BRWD3 and H3K4 methylation, however, has yet to be defined. Here, we show that depleting BRWD3 not only causes an increase in H3K4me1 levels but also causes a decrease in H3K4me3 (H3 lysine 4 trimethylation) levels, indicating that BRWD3 influences H3K4 methylation more broadly. Using immunoprecipitation coupled to quantitative mass spectrometry, we identified an interaction between BRWD3 and the H3K4-specific lysine demethylase 5 (KDM5/Lid), an enzyme that removes tri- and dimethyl marks from H3K4. Moreover, analysis of ChIP-seq (chromatin immunoprecipitation sequencing) data revealed that BRWD3 and KDM5 are significantly colocalized throughout the genome and H3K4me3 are highly enriched at BRWD3 binding sites. We show that BRWD3 promotes K48-linked polyubiquitination and degradation of KDM5 and that KDM5 degradation is dependent on both BRWD3 and Cul4. Critically, depleting KDM5 fully restores altered H3K4me3 levels and partially restores H3K4me1 levels upon BRWD3 depletion. Together, our results demonstrate that BRWD3 regulates KDM5 activity to balance H3K4 methylation levels.

## Significance

H3K4 methylation is linked to active gene transcription, but mechanisms controlling H3K4me3 (H3 lysine 4 trimethylation) levels remain unclear. Here, we investigate the role of BRWD3 (Bromodomain and WD repeat-containing protein 3), a chromatin-binding protein and substrate-specificity factor for the Cul4^DDB1^ ubiquitin ligase complex, in regulating H3K4 methylation. We show that depletion of BRWD3 not only increases H3K4me1 levels as previously reported but also decreases H3K4me3 levels. We find that BRWD3 associates with KDM5/Lid, a demethylase that primarily removes tri- and dimethyl marks from H3K4 and that BRWD3 promotes KDM5 degradation through K48-linked polyubiquitination. Codepletion of KDM5 restores H3K4me3 levels associated with BRWD3 depletion. Our findings identify BRWD3 as a critical factor in regulating the balance of H3K4 methylation levels.

Epigenetic regulation of gene expression through histone modifications can alter chromatin structure and function without any alteration in DNA sequence. This allows for changes in gene expression that can ultimately drive cell differentiation and disease states (1, 2). The N-terminal tails of histones undergo numerous modifications including methylation, acetylation, and phosphorylation (3). These modifications affect chromatin structure and serve as binding platforms for transcription factors, chromatin remodelers, and other regulatory proteins (3).

One histone modification that is commonly associated with actively transcribed genes is histone H3 lysine 4 methylation (1, 4, 5). H3K4 can be methylated up to three times, resulting in mono-, di-, or trimethylation (H3K4me1, H3K4me2, or H3K4me3, respectively) (6). While H3K4me3 is primarily enriched at gene promoters and around transcription start sites, H3K4me2 is spread throughout intragenic and intergenic regions, and H3K4me1 is commonly found at enhancer regions (7–9). Normal H3K4 methylation patterns are important for development, and the steady state of H3K4 methylation is controlled by a balance between H3K4-specific lysine methyltransferases (KMTs) that add methyl groups and H3K4-specific lysine demethylases (KDMs) that remove methyl groups (6, 10). The Set1-domain containing complex, known as COMPASS (Complex of Proteins Associated with Set1), catalyzes mono-, di-, and trimethylation of histone H3K4 (6). On the other hand, there are two families of demethylases, KDM1 and KDM5, that remove methyl groups from H3K4. While the KDM1 family of enzymes removes methyl groups from H3K4me1 and H3K4me2, the KDM5 family preferentially removes the methyl group from H3K4me3 and to a lesser extent H3K4me2 (11–14). The enzymes that control H3K4 methylation are essential for normal cellular function (4). For example, altered expression of human *KDM5* genes has been implicated in various cancers, and *KDM5* mutations are linked to intellectual disorders (15–18). Although the enzymes that control H3K4 methylation are well defined, how these enzymes are regulated to balance H3K4 methylation status is largely unknown.

The evolutionary conserved protein, Bromodomain and WD repeat-containing protein 3 (BRWD3), binds directly to H3K4 methylation in vitro through a cryptic Tudor domain (19). The BRWD3 homolog also has two bromodomains that bind to acetylated histones with simultaneous binding of H3K4 methylation (20). Interestingly, loss of BRWD3 function causes an increase in H3K4me1 levels in Drosophila, but the underlying mechanism is completely unknown (19). BRWD3 functions as a substrate receptor of the Cul4 E3 ubiquitin ligase complex (21–23). While Cul4 is known to target many DNA replication and cell cycle proteins for degradation, how BRWD3 contributes to epigenetic maintenance and if ubiquitin targeting activity is involved is not clear (21, 24, 25). Mutations in the *BRWD3* family of genes were recently identified as the cause of a neurodevelopmental disorder, and altered *BRWD3* expression has been found in various cancers (26–30). Thus, defining the molecular function of BRWD3 in epigenetic maintenance could be an important step for understanding the molecular causes of disease states and identifying potential therapeutic targets.

Drosophila is an ideal system to understand how BRWD3 regulates H3K4 methylation (31). Drosophila has a single BRWD3 homolog, whereas humans have three BRWD3 orthologs (BRWD1, BRWD2, and BRWD3). Furthermore, H3K4 methylation is streamlined in Drosophila relative to humans. Drosophila has three H3K4 methyltransferase complexes (Set1/COMPASS, Tri, and Trr), whereas humans have six (SET1A, SET1B, MLL1, MLL2, MLL3, and MLL4) (6). For H3K4me3/me2 demethylation, Drosophila has a single KDM5 family member (KDM5/Lid), whereas humans have up to four KDM5 orthologs (KDM5A, KDM5B, KDM5C, and KDM5D) (16).

In this study, we show that BRWD3 has a global effect on H3K4 methylation levels. While loss of BRWD3 function has been shown to promote an increase in H3K4me1 levels, we find that H3K4me3 levels are reduced upon depletion of BRWD3. Strikingly, we identify KDM5 as a BRWD3-associated protein. In vivo ubiquitination assays reveal that BRWD3 promotes K48-linked polyubiquitination on KDM5. Furthermore, KDM5 is rapidly degraded in a BRWD3- and Cul4-dependent manner. Critically, codepletion of KDM5 can restore H3K4me3 levels upon BRWD3 depletion. We also find that *BRWD3* is a *su(var)* gene and loss of a single copy of *KDM5* can suppress the *BRWD3* Su(var) phenotype. Taken together, our work supports a model in which BRWD3 controls the stability of KDM5 to regulate demethylation of H3K4me3 and maintain overall H3K4 methylation levels.

## Results

### BRWD3 Affects H3K4 Mono-, Di-, and Trimethylation Levels.

**Fig. 1.**
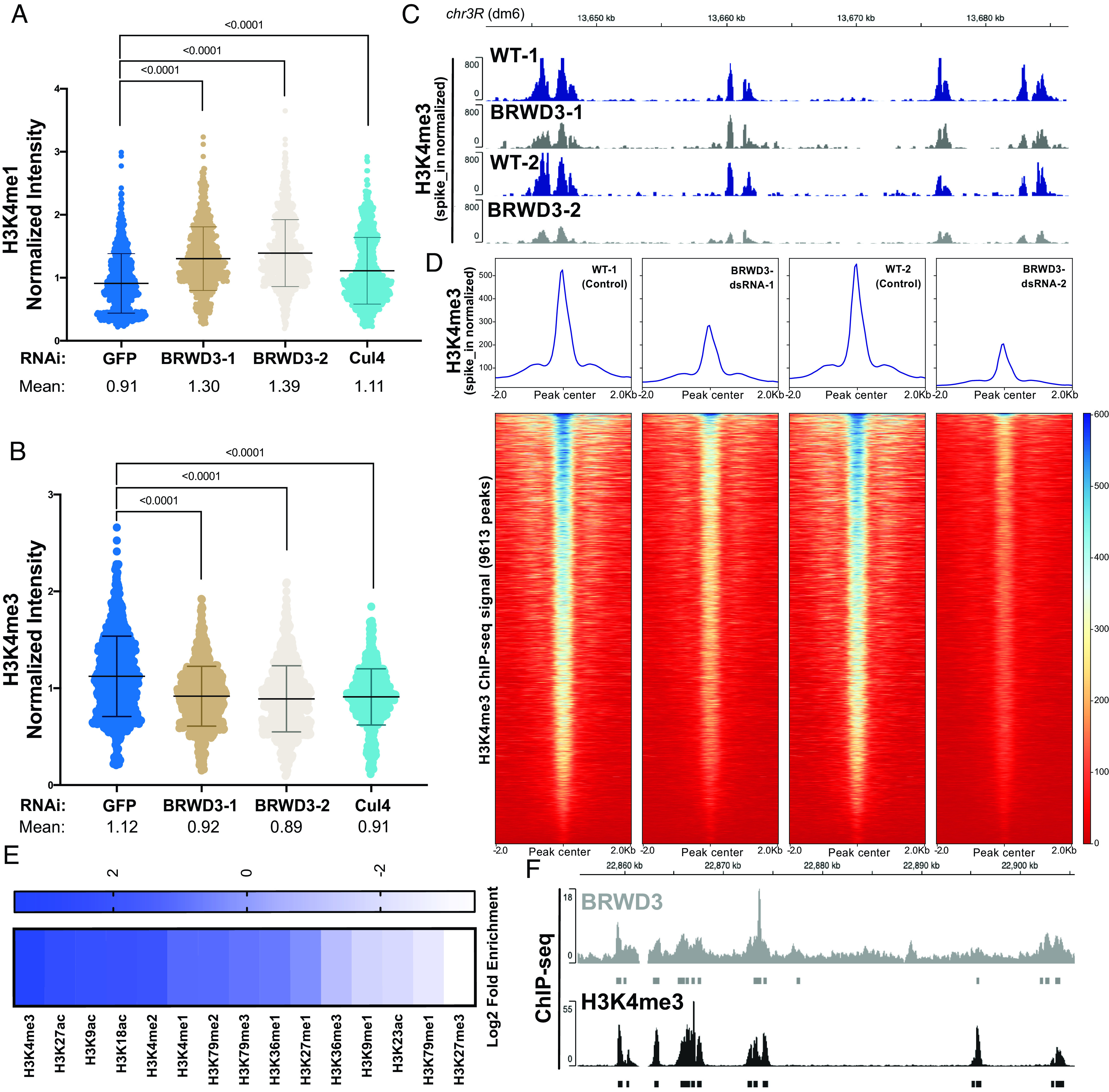
BRWD3 affects H3K4 methylation status. Quantification of H3K4me1 (*A*) and H3K4me3 (*B*) intensity using quantitative IF in Drosophila S2 cells. Each dot presents the H3K4 methylation intensity per nucleus normalized to the total DNA content. Each distribution represents the signal intensities of 1,000 randomly selected cells from three biological replicates. *P* < 0.0001 using an ANOVA one-way analysis with Tukey's multiple comparisons test. (*C*) Representative H3K4me3 spike-in normalized ChIP-seq profiles generated in S2 cells with no dsRNA control (WT) and BRWD3 depletion using two independent dsRNAs (*Methods*). (*D*) Enrichment heatmap of spike-in normalized H3K4me3 ChIP-seq signal sorted by mean occupancy around the centers of all published H3K4me3 peaks. (*E*) Enrichment of H3 marks within BRWD3 binding sites. Log2 enrichment for observed overlap relative to expected overlap for each mark peak set is shown. (*F*) Representative view showing the similarity of BRWD3 and H3K4me3 ChIP-seq tracks. Data were from ref. 19.

In Drosophila, BRWD3 is required to maintain H3K4me1 levels, although the mechanism is unknown (19). Due to the dynamic regulation of H3K4me1, me2 and me3, we were curious whether BRWD3 also influenced H3K4me3 and/or me2 methylation levels. In addition, we wanted to determine whether BRWD3 control of H3K4 methylation is dependent on Cul4 E3 ubiquitin ligase given that BRWD3 is a substrate specificity factor for the Cul4 ubiquitin ligase complex (21). To measure the relative level of H3K4 methylation marks, we depleted BRWD3 and Cul4 in Drosophila S2 cells and measured H3K4me1, me2, and me3 levels by quantitative immunofluorescence (IF). Consistent with previous results, BRWD3 depletion caused an increase in H3K4me1 levels relative to negative control cells Green Fluorescent Protein (GFP) (Fig. 1*A*). We found that BRWD3 depletion caused a decrease in H3K4me3 levels (Fig. 1*B*) and an increase in H3K4me2 levels (*SI Appendix*, Fig. S1*A*). Consistent with the quantitative IF results, western blot analysis showed the same trend for H3K4me1 and me3 levels, with a significant increase in H3K4me1 levels and a decrease in H3K4me3 levels upon BRWD3 depletion (*SI Appendix*, Fig. S1*C*). Importantly, Cul4 depletion resulted in similar changes in H3K4me1 (Fig. 1*A*), me2 (*SI Appendix*, Fig. S1*A*), and me3 levels (Fig. 1*B*). Taken together, these results demonstrate that BRWD3 affects pan H3K4 methylation levels and likely does so in a ubiquitination-dependent manner.

To further test whether BRWD3 regulates H3K4me3 levels using an independent approach, we reanalyzed previously published H3K4me3 ChIP-seq data generated in control (lacZ) and BRWD3-depleted Drosophila S2 cells (19). To quantify the change in published H3K4me3 ChIP-seq read counts, we centered all H3K4me3 peaks and quantified the read counts for each peak. We found that BRWD3 depletion caused a ~50% reduction of H3K4me3 ChIP-seq read counts (*SI Appendix*, Fig. S1*B*). While consistent with our IF and western blot data, these ChIP-seq datasets did not contain spike-in controls for normalization. Therefore, we performed ChIP-seq of H3K4me3 with and without BRWD3 depletion using SNAP synthetic nucleosomes for spike-in normalization (EpiCypher, see *Methods*) (32). Using spike-in normalized data, we found that depletion of BRWD3 caused a reduction H3K4me3 ChIP-seq reads at H3K4me3 peak regions relative to negative control cells (Fig. 1*D*). The reduction of H3K4me3 signal was uniform throughout the genome (Fig. 1*C*). To validate these ChIP-seq results, we conducted ChIP-qPCR analysis of the same spike-in ChIP reactions. These data demonstrate that BRWD3 depletion caused a decrease in H3K4me3 signal at known H3K4me3 sites but not at negative control sites (*SI Appendix*, Fig. S1 *E* and *F*). Based on our ChIP-seq, ChIP-qPCR, IF, and western blot data, we conclude that BRWD3 regulates not only H3K4me1 but also H3K4me2 and H3K4me3 levels.

BRWD3 homologs have a cryptic Tudor domain that is critical for binding to methylated H3 and two bromo domains necessary for binding to acetylated H3 (19). The human BRWD2 homolog PHIP binds to both H3K4 methylated histones and acetylated histone H3 peptides in vitro (19, 20). Given that BRWD3 affects H3K4me1, me2, and me3 levels in vivo, we wanted to determine whether BRWD3 associates with chromatin containing these specific histone modifications throughout the genome. To this end, we evaluated the overlap between BRWD3 and all histone modifications available through the modENCODE consortium (33, 34). For each dataset, we compared the observed overlap with the expected overlap obtained from 1,000 randomly shuffled sets of peaks (35, 36). This allowed us to calculate both the enrichment and significance of any overlap. Overall, active histone modifications were significantly enriched around BRWD3 binding sites, while repressive histone marks were significantly depleted, consistent with previous observations that BRWD3 is associated with active histone marks (Fig. 1*E*) (19). Interestingly, H3K4me3 was the most significantly enriched mark at BRWD3 binding sites among all tested H3 modifications (*P* value = 0.001, log2-fold change = 3.48) (Fig. 1 *E* and *F*). Importantly, H3K4me3 was enriched at a higher level than H3K4me2 and H3K4me1, which mirrors the in vitro binding affinity of the BRWD3 cryptic Tudor domain for these marks (Fig. 1*E*) (19, 20). Additionally, H3K27ac, H3K9ac, and H3K18ac were also enriched in BRWD3 binding sites, consistent with the BRWD3 bromodomain binding to these modifications in combination with H3K4 methylation in vitro (20). Together, these data suggest that while BRWD3 associates with methylated H3K4 in vitro, BRWD3 preferentially associates with H3K4me3 in vivo.

### BRWD3 Associates with the H3K4me3 Histone Demethylase KDM5/Lid.

**Fig. 2.**
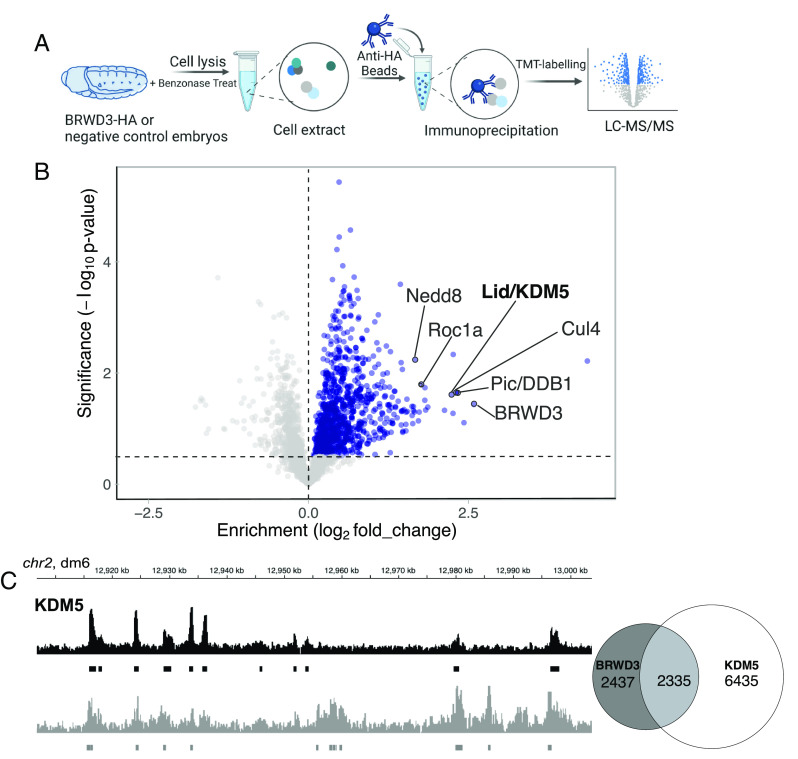
BRWD3 and KDM5 are in the same complex and bind the same genomic regions. (*A*) Workflow of BRWD3-HA IPs from Drosophila embryos followed by mass spectrometry. (*B*) Volcano plot of the BRWD3-IP-TMT-MS results. Blue dots represent hits significantly enriched in BRWD3-HA-IP compared to the negative control. The red dots represent the Cul4-DDB1-BRWD3 E3 ubiquitin ligase complex. (*C*) Representative view showing the similarity of BRWD3 and KDM5 ChIP-seq tracks (*Left*). Venn diagram quantifying the overlap between BRWD3 and KDM5 binding sites on the genome, *P* < 0.0001 (*Right*).

H3K4 methylation levels are controlled by both histone methyltransferase and histone demethylase activities (6). To explore the mechanism by which BRWD3 affects H3K4 methylation status, we used immunoprecipitation (IP) followed by quantitative mass spectrometry to identify BRWD3 interacting proteins that could potentially regulate H3K4 methylation levels. To this end, we constructed an endogenously HA-tagged BRWD3 fly line using the CRISPR-Cas9-based knock-in method (37, 38). Loss of BRWD3 function is lethal in Drosophila (39, 40). The endogenously tagged *BRWD3-HA* homozygous line, however, produced a protein of the expected size and was viable indicating that BRWD3-HA is functional (*SI Appendix*, Fig. S2). We immunoprecipitated BRWD3-HA from Benzonase-digested embryo extracts, labeled IP material with tandem mass tags (TMT), and identified BRWD3-interacting proteins by tandem qMS (Fig. 2*A* and Dataset S1). We observed a significant enrichment of Cul4, Pic/DDB1, and Roc1A that are known components of the Cul4 E3 ubiquitin ligase complex (Fig. 2*B*) (23). Interestingly, we also identified Nedd8, which covalently modifies Cul4 to activate Cul4 ubiquitin ligase activity, indicating that BRWD3 can associate with activated Cul4 (Fig. 2*B*) (41). Strikingly, we identified the H3K4-specific lysine demethylase KDM5/Lid as a BRWD3-interacting protein (Fig. 2*B*).

Given the association between BRWD3 and KDM5, we next asked whether BRWD3 and KDM5 share similar binding sites on chromatin. To this end, we analyzed previously published ChIP-seq data for BRWD3 (from Drosophila S2 cells) and KDM5 (from Drosophila adults) (19, 42). Even though these datasets are from disparate developmental samples, we found that ~50% of BRWD3 binding sites overlap with KDM5 sites (Fig. 2*C*). Taken together, we conclude that BRWD3 binding sites show significant overlap with KDM5 sites genome wide. Given that KDM5 can sequentially remove the methyl group from H3K4me3 to convert it to H3K4me2 and further to H3K4me1, we hypothesize that BRWD3 influences H3K4me3 levels by regulating KDM5 activity (13, 43).

### BRWD3 Promotes KDM5 Ubiquitination and Degradation.

**Fig. 3.**
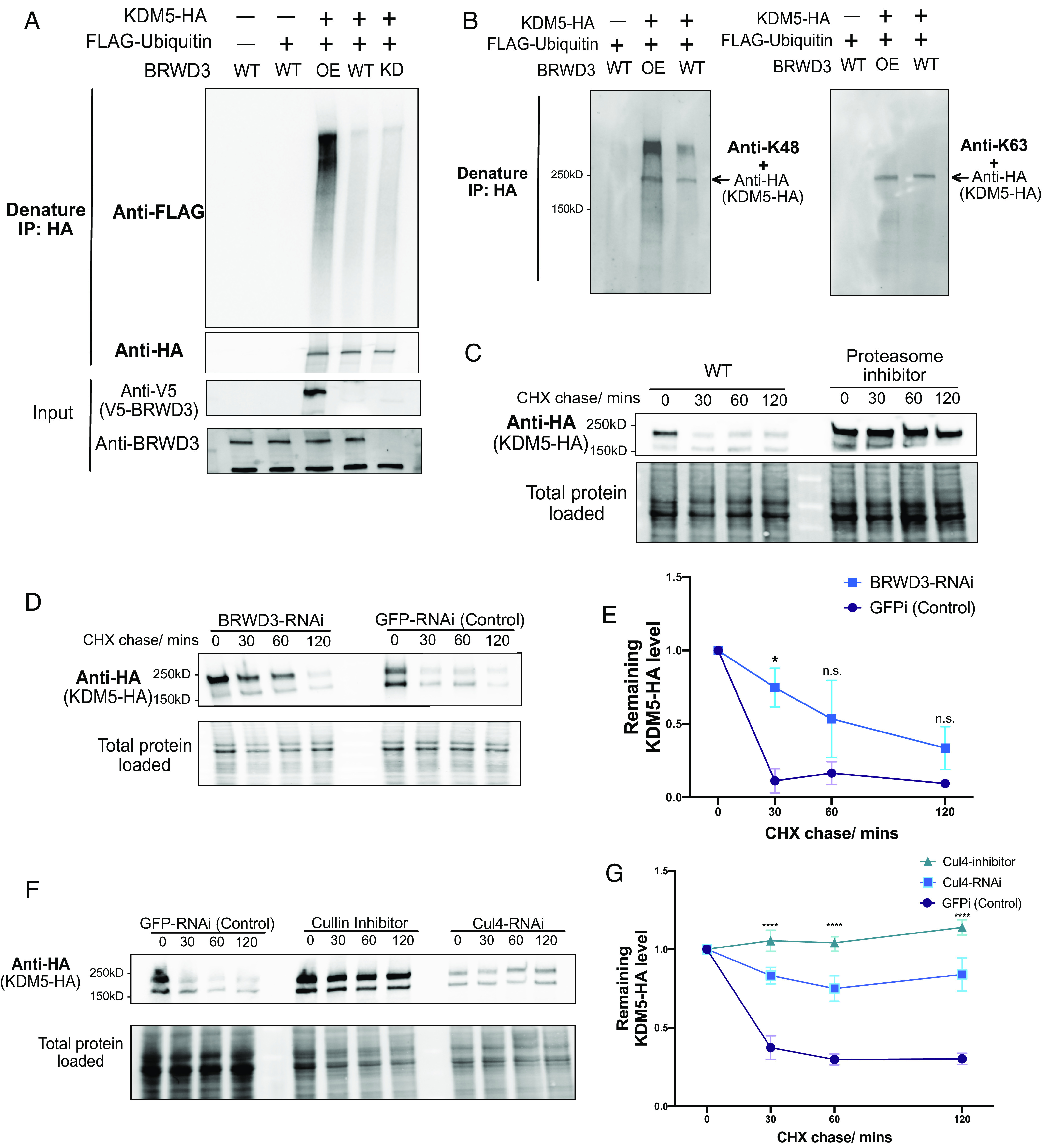
BRWD3 promotes KDM5 ubiquitination and degradation. (*A*) Denaturing IPs from Drosophila S2 cells cotransfected with HA-tagged KDM5 and/or FLAG-tagged ubiquitin in BRWD3-V5 cotransfected (OE), wild-type (WT), or BRWD3 RNAi (KD) conditions. (*B*) Immunoprecipitates from denaturing IPs were blotted with antibodies specific for K48- or K63-linked polyubiquitin chains together with anti-HA antibody. (*C*) S2 cells were transfected with plasmids expressing KDM5-HA and treated with cycloheximide (CHX) only or together with the proteasome inhibitor MG-132. After the indicated time with CHX treatment, western blots with anti-HA antibody were performed to measure the KDM5-HA level. (*D*) Similar as *C*, but with either *BRWD3*-RNAi or *GFPi*-RNAi treatment. (*E*) Quantification of the KDM5-HA level from three biological replicates of (*D*) at the indicated time points normalized to the total loaded protein level. (*F*) Similar to *C* in S2 cells with either *GFPi*-RNAi, *Cul4*-RNAi, or treatment with the Cullin inhibitor (MLN4924). (*G*) Quantification of the remaining KDM5-HA levels from three biological replicates of *F* at the indicated time points.

Our results show that BRWD3 associates with KDM5 and is required for normal H3K4me3 levels. Given that BRWD3 is a substrate specificity factor for the Cul4 E3 ubiquitin ligase, BRWD3 could control H3K4me3 levels by targeting KDM5 for ubiquitination and possibly degradation. Thus, upon depletion of BRWD3, KDM5 levels may become elevated resulting in excessive H3K4me3 demethylase activity. To test this hypothesis, we first assessed whether KDM5 ubiquitination was dependent on BRWD3 in vivo (*Methods*). In these experiments, we coexpressed HA-tagged KDM5 with FLAG-ubiquitin in Drosophila S2 cells and analyzed KDM5 ubiquitination status following an HA-IP under denaturing conditions. We detected a low level of ubiquitination of KDM5 in control cells (Fig. 3*A*). BRWD3 depletion, however, did not cause a significant effect on KDM5 ubiquitination, which would be expected if multiple ubiquitin ligases targeted KDM5 (Fig. 3*A*). In contrast, overexpression of BRWD3 greatly enhanced KDM5 ubiquitination (Fig. 3*A*). By using ubiquitin-linkage-specific antibodies, we found that BRWD3 promotes K48-linked polyubiquitination of KDM5 (Fig. 3*B*). We conclude that BRWD3 promotes K48-linked ubiquitination of KDM5, suggesting that BRWD3 could affect KDM5 stability.

To directly test whether BRWD3 affects KDM5 stability, we used a cycloheximide (CHX) chase assay to measure the half-life of KDM5. CHX inhibits new protein synthesis by blocking translation elongation (44). This allows for the measurement of protein degradation given that new protein synthesis is blocked. Interestingly, we found that KDM5 was rapidly degraded with a half-life of less than 30 min (Fig. 3*C*). Importantly, KDM5 levels could be stabilized by adding the proteasome inhibitor MG-132, demonstrating that KDM5 degradation is proteasome-dependent (Fig. 3*C*). Next, we tested whether the degradation of KDM5 was dependent on BRWD3. We found that BRWD3 depletion slowed the degradation of KDM5 relative to negative control (Fig. 3 *D* and *E*). KDM5 levels, however, were only partially stabilized upon depletion of BRWD3 and were reduced to background levels in ~120 min (Fig. 3 *D* and *E*).

Since we did not find significant change in KDM5 ubiquitination upon BRWD3 depletion, we reasoned that other E3 ubiquitin ligases could also target KDM5 to control its stability (Fig. 3*A*). To test whether KDM5 is degraded in a Cul4-dependent manner, we inhibited Cul4 activity by RNAi or by adding the Cullin-specific inhibitor MLN4924 (45). We found that Cul4 inhibition slowed KDM5 degradation to a greater extent than BRWD3 depletion (Fig. 3 *F* and *G*). At 120 min, the remaining KDM5 levels in Cul4 depletion were much higher than those in BRWD3 depletion, suggesting that other Cul4 substrate receptors can target KDM5 for ubiquitination (Fig. 3 *F* and *G*). Taken together, we conclude that BRWD3 promotes K48-linked polyubiquitin of KDM5 and likely in a Cul4-dependent manner to direct KDM5 degradation.

### Depleting KDM5 Restores Altered H3K4me3 Levels upon BRWD3 Depletion.

**Fig. 4.**
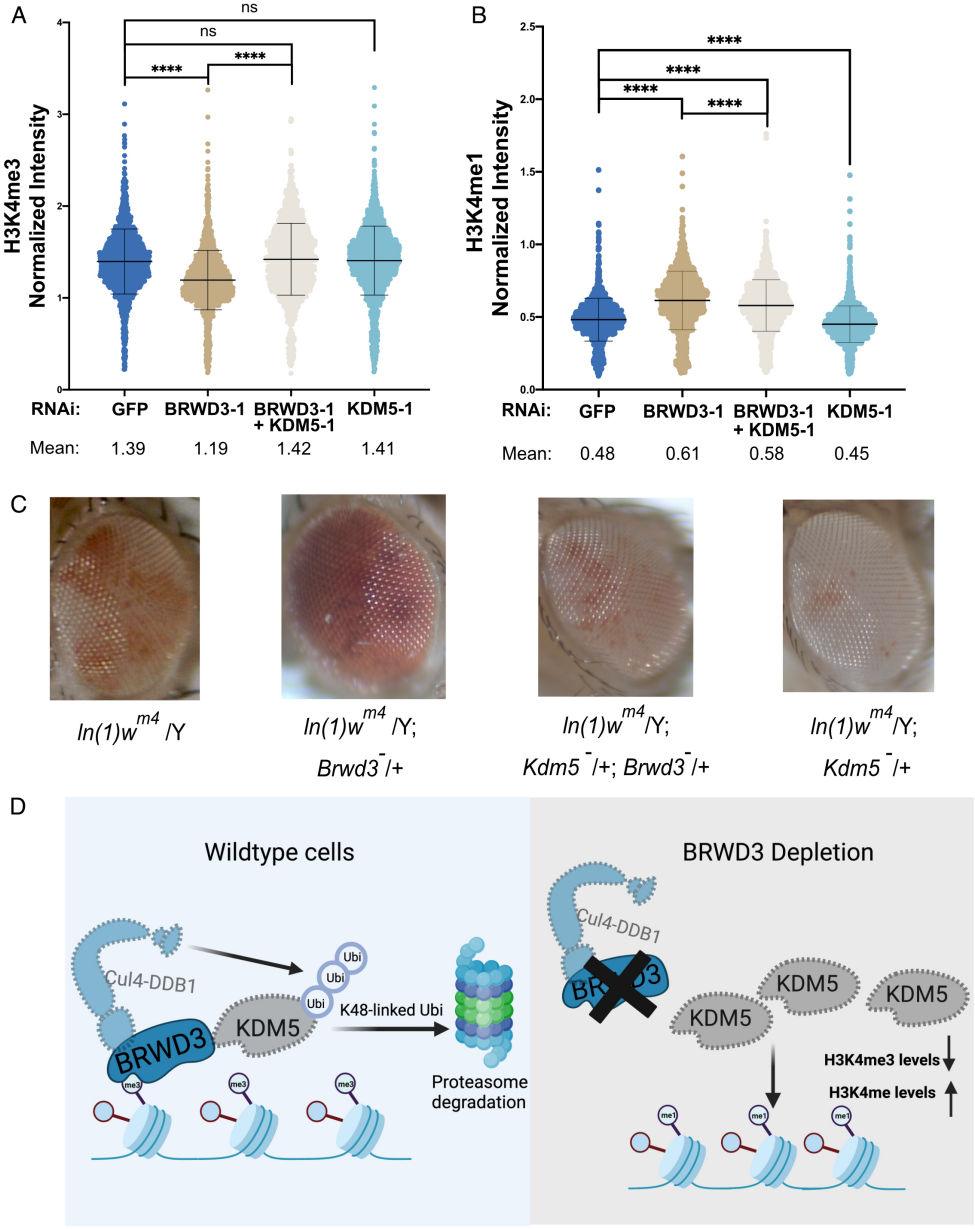
Inhibiting KDM5 restores altered H3K4 methylation levels upon BRWD3 depletion. (*A*) Violin plot of quantitative IF assays in Drosophila S2 cells using an anti-H3K4me3 antibody (*A*) or an anti-H3K4me1 antibody (*B*). Five micrograms of KDM5 dsRNA was used in codepletion. Each distribution represents the signal intensities of 1,000 randomly selected cells from three biological replicates. ****P < 0.0001 using the ANOVA one-way analysis with Tukey’s multiple comparisons test. (*C*) Loss of a single copy of *BRWD3* gives a Su(var) phenotype that is dependent on *KDM5.* (*D*) A model for BRWD3-dependent regulation of H3K4 methylation levels. BRWD3 targets KDM5 for degradation. In the absence of BRWD3, KDM5 activity is increased resulting in demethylation of H3K4me3, which generates increased H3K4me1 levels.

Given that BRWD3 controls KDM5 stability, we predict that KDM5 is overactive in BRWD3-depleted cells. In vitro, KDM5 demethylases preferentially target H3K4me3 and to a lesser extent H3K4me2 with little to no activity on H3K4me1 (13, 43). Therefore, we predict that overactive KDM5 removes methyl groups from H3K4me3, ultimately resulting in increased H3K4me1 levels and explaining the altered levels of H3K4 methylation in BRWD3 depleted cells. To test this prediction, we codepleted KDM5 in BRWD3-depleted S2 cells and measured the relative H3K4me3 by quantitative IF. We treated cells with a low dose of KDM5 dsRNA to limit changes in H3K4me3 levels (*SI Appendix*, Fig. S4*A*). Using a dsRNA concentration that had only a minimal impact on H3K4me3 levels in unperturbed S2 cells, we were able to restore H3K4me3 levels upon BRWD3 depletion (Fig. 4*A*). To confirm this result, we codepleted BRWD3 and KDM5 with an independent set of dsRNAs and found that H3K4me3 levels were also restored (*SI Appendix*, Fig. S4*C*). To further test our prediction, we measured H3K4me1 levels in BRWD3 and KDM5 codepleted cells. While we did not observe a full restoration, H3K4me1 levels were significantly reduced upon KDM5 codepletion (Fig. 4*B*) (*SI Appendix*, Fig. S4*B*). It is known that BRWD3 associates with chromatin and plays key roles in regulating gene expression and histone modifications (19). Our data indicate that KDM5 is a critical mediator of BRWD3-dependent changes in histone methylation. We wanted to test whether BRWD3 affects chromatin architecture in vivo and, if so, whether this activity is dependent on KDM5 function. Many genes that regulate chromatin structure and function have been identified as modifiers of position effect variegation (PEV) (46). Interestingly, we found that *BRWD3* dominantly suppressed PEV. Loss of one copy of *BRWD3* resulted in an increase in variegated *white* expression relative to control flies (Fig. 4*C*). Thus, we conclude that BRWD3 is a *Su(var)* gene. Critically, loss of a single copy of *KDM5* suppressed the *BRWD3* Su(var) phenotype, whereas deleting one copy of KDM5 itself led to the suppression of PEV compared to control flies (Fig. 4*C*). Consistent with our work in S2 cells, this indicates that the ability for BRWD3 to affect H3K4 methylation levels and/or function depends on KDM5.

Our model predicts that BRWD3 protects H3K4me3 from demethylation by targeting KDM5 for degradation. To test this prediction, we analyzed H3K4me3 levels at sites where KDM5 coexists with BRWD3 compared to KDM5 sites only (as determined by ChIP-seq). Our findings indicate that H3K4me3 levels are higher at sites where BRWD3 and KDM5 colocalize, suggesting that BRWD3 could safeguard H3Kme3 levels throughout the genome (*SI Appendix*, Fig. S4*C*). Taken together, we propose that BRWD3 promotes K48-linked polyubiquitin of KDM5 to promote KDM5 degradation, thus maintaining normal H3K4 methylation levels (Fig. 4*D*).

To test whether changes in steady-state RNA levels upon BRWD3 depletion are correlated with changes in H3K4me3 levels, we analyzed previously published RNA-seq datasets comparing BRWD3-depleted and wild-type (WT) S2 cells (19). We found that a similar number of transcripts are down-regulated (1,175) or up-regulated (1,145) upon BRWD3 depletion, with the majority of transcripts (8,170) remaining unchanged (Dataset S3). Next, we mapped the H3K4me3 ChIP-seq signal (spike-in normalized) around these three distinct groups of gene regions. Our analysis revealed a global reduction in H3K4me3 levels regardless of changes in transcript levels (*SI Appendix*, Fig. S4*E*).

## Discussion

Depletion of BRWD3 results in increased H3K4me1 levels (19). The mechanism connecting BRWD3 and H3K4 methylation, however, had yet to be defined. Through several independent approaches, we have found that BRWD3 targets KDM5, a demethylase that targets H3K4me2/me3, for ubiquitination and ultimate proteasomal-dependent degradation. By targeting KDM5, BRWD3 influences not only H3K4me1 but H3K4 di- and trimethylation. Thus, BRWD3 is a key regulator of pan H3K4 methylation levels. Although we cannot yet conclude that BRWD3 directly targets KDM5 for polyubiquitination, depletion of BRWD3 or Cul4 can stabilize KDM5, indicating that KDM5 polyubiquitination is likely directed by Cul4^BRWD3^. In support of this idea, codepleting KDM5 upon BRWD3 depletion fully restores changed H3K4me3 levels and partially restores H3K4me1 levels upon BRWD3 depletion. Therefore, we propose that BRWD3 targets KDM5 for degradation to maintain the normal H3K4 methylation levels.

We found that KDM5 is unstable, with a half-life of ~30 min in Drosophila. We also found that Cul4^BRWD3^ is not the only ubiquitin ligase that targets KDM5 for ubiquitination and ultimate proteasomal-dependent degradation. This suggests that KDM5 activity is highly regulated and KDM5 is likely subject to extraordinary regulation to maintain H3K4 methylation levels. In yeast and human cells, the E3 ubiquitin ligase Not4 has been shown to target KDM5 homologs for polyubiquitination and degradation (47). It is unknown, however, whether Not4 targets KDM5 for ubiquitination in Drosophila. Given that inhibition of Cul4 results in a near-complete stabilization of KDM5, it is likely that Cul4 uses other substrate receptors to target KDM5 for polyubiquitination in Drosophila. H3K4me3 is associated with actively transcribed genes and can enhance binding of transcription factor II D to promoters (47, 48). Recent evidence suggested that H3K4me3 has a role in regulating RNA polymerase II promoter-proximal pause-release (43). H3K4me1, on the other hand, is enriched at enhancers and may serve to recruit chromatin-modifying proteins to enable enhancer activity (7, 48). Therefore, carefully regulating the balance of H3K4 methylation states is likely important for gene expression changes. Recent work in mammalian cells has revealed that KDM5 homologs can eliminate nearly all H3K4me3 in less than 2 h (43). Given the potential for such rapid demethylation, KDM5 would be an attractive target for extensive posttranslational regulation (43). Control of KDM5 activity could be critical for regulating the gene expression programs that occur during cell differentiation. Thus, identifying the additional factors that target KDM5 for degradation will be important to understand how KDM5 activity is regulated during development and disease.

Out of all the histone modifications we could assay using an unbiased computational approach, we found that H3K4me3 was the most significantly enriched histone modification at BRWD3 binding sites. This is consistent with previous in vitro data demonstrating that BRWD3 preferentially binds to H3K4me3 (19, 20). Given that KDM5 is a primary demethylase for H3K4me3/me2, it is possible that BRWD3 binds to H3K4me3 on chromatin and protects H3K4me3 by blocking KDM5 occupancy and targeting KDM5 for degradation. In this case, BRWD3 could form a local zone of protection for H3K4me3, thus increasing the half-life of this mark. In human cells, BRWD3 is necessary to recruit Cul4 onto chromatin. Therefore, it seems likely that the BRWD3-dependent degradation of KDM5 happens in the context of chromatin. Upon BRWD3 depletion, Cul4 would not be properly recruited to chromatin and KDM5 activity would go unchecked, resulting in a ubiquitous decrease in H3K4me3 levels throughout the genome. This is consistent with our findings demonstrating that global levels of H3K4me3 are reduced upon BRWD3 depletion. KDM5 removes methyl groups from H3K4me3 in a sequential manner, first converting H3K4me3 to H3K4me2 and then H3K4me2 to H3K4me1 (13). In vitro, KDM5 primarily targets H3K4me3, and to a lesser extent H3K4me2, with limited activity on H3K4me1 (13, 49–51). Recent in vivo time-course assays found that KDM5 enzymes can demethylate H3K4me3 in less than 2 h but require nearly 24 h to demethylate H3K4me2 or H3K4me1 (43). This supports the finding that KDM5 preferentially targets H3K4me3 in vitro (13). We suspect that the unchecked KDM5 activity upon BRWD3 depletion results in the rapid demethylation of H3K4me3, disrupting the balance of H3K4 methylation and shifting H3K4 methylation toward H3K4me1 at the expense of H3K4me3.

To test our model, we demonstrate that codepletion of KDM5 can fully suppress the decreased H3K4me3 levels and partially suppress the increased H3K4me1 levels upon BRWD3 depletion. While the H3K4me1 levels were not completely restored upon KDM5 codepletion, trying to precisely reduce the KDM5 levels with RNAi given the kinetics of H3K4 methylation/demethylation complicate this experiment. Nonetheless, together with all of our data, these results suggest that increased KDM5 activity upon BRWD3 depletion drives changes in pan H3K4 methylation levels. Multiple human genetic studies have demonstrated that human *BRWD3* and *KDM5C* mutations underlie X‐linked intellectual disability (17, 18, 29, 30). Further, higher *BRWD3* mutation rates have been found in cancers and linked to a decrease in overall survival. Unfortunately, the underlying mechanisms resulting in disease in these cases remain unknown (52, 53). Interestingly, overexpression of KDM5A or KDM5B is also implicated in the progression and drug resistance of several cancers (16, 54–56). While it is unknown that whether BRWD3 or any of its orthologs promotes KDM5 homolog degradation in humans, it is intriguing to consider that mutations in *BRWD3* could cause increased levels of KDM5. If this is the case, then KDM5 inhibitors could be a potential treatment for *BRWD3*-associated diseases, although further studies will be required to determine whether this pathway is conserved.

## Methods

### RNA Interference.

RNAi in S2 cells was performed following previously published methods (57). Briefly, dsRNAs were synthesized using the Invitrogen MEGAscript T7 Transcription Kit (Ambion). A list of primers used to generate dsRNA can be found in Dataset S2. For each sample, 1.5 million S2 cells were seeded in 1 mL nonserum medium in a six-well plate. Five to twenty micrograms of dsRNA was added and incubated for 45 min at room temperature, and then, 2 mL of serum-containing medium was added. Cells were incubated for an additional 4 d. To confirm the efficiency of RNAi depletion, ~3 million cells were harvested, and proteins were extracted by boiling in 2× Laemmli sample buffer (Bio-Rad). Protein levels were measured by western blotting with antibody (Rabbit anti-BRWD3, 1:500).

### Plasmid Construction.

Plasmid pMT (metallothionein promoter)-BRWD3-V5 was created by inserting the BRWD3 CDS sequence into the pMT-puro vector (Addgene #17923) using the Gibson assembly method provided by New England Biolabs (NEB) (# E5510S). For the pUbi63E-Flag-Ubiquitin plasmid, a gene block consisting of the pUbi63E promoter and Flag-Ubiquitin CDS sequence was chemically synthesized by Twist Biosciences. Subsequently, the gene block was integrated into the pMT-puro vector by replacing the existing pMT promoter and V5 tag sequences. Finally, the pUbi63E-KDM5-HA plasmid was generated by replacing the Flag-Ubiquitin CDS sequence with the KDM5-HA CDS sequence using Gibson assembly technology provided by NEB (# E5510S).

### Fly Stocks and PEV Assays.

The following lines were acquired from the Bloomington Stock Center: *ln*(1)*white m^4^*(BDSC#32578), *BRWD3^PX1^*(BDSC #35560), and *lid*/*kdm5^10424^* (BDSC # 12367). ORR (Oregon-R) flies were a gift from Terry Orr-Weaver (Whitehead Institute). Endogenously HA-tagged *BRWD3-HA* fly line was constructed using the CRISPR/Cas9-catalyzed homology-directed knock-in method following the online protocol (https://flycrispr.org/scarless-gene-editing/). The 2× HA tag was inserted in the C terminus of BRWD3 protein. The plasmid injections were performed by BestGene Inc. (https://www.thebestgene.com). For PEV assays, *ln*(1)*white m^4^*exhibits variegation of red and white eye color and was used as a control for the experiments. *ln*(1)*white m^4^* virgins were crossed to *BRWD3^PX1^* males and their male offspring were examined for eye color. *ln*(1)*white m^4^*/*w^−^*; *BRWD3^PX1^*/ *TM3* was crossed to male *kdm5^10424^*/*CyO* and their male offspring were examined for the eye color.

### Quantitative IF.

RNAi-treated cells were attached to Concanavalin A–coated slides for 15 min, fixed for 15 min in 4% paraformaldehyde (PFA), and permeabilized for 15 min in Phosphate-buffered Saline (PBS) supplemented with 0.3% Triton-X-100 Phosphate-Buffered Saline with Triton X-100 (PBT). Cells were then blocked for 60 min in blocking buffer (1% Bovine Serum Albumin and 0.2% goat serum in 0.1% PBT). After blocking, cells were incubated with rabbit anti-H3K4me1 (Cell Signaling #5326, 1:1,000), anti-H3K4me2 (Cell Signaling #9725, 1:1,000), or anti-H3K4me3 (Active Motif # 39060, 1:1,000) antibodies overnight at 4 °C in blocking buffer. After washing with PBT, cells were incubated with goat Alexa fluorophore 568–conjugated anti-rabbit IgG secondary antibody (1:500, Life Technologies, # A11011) in blocking buffer for 1 h at room temperature. After three washes with PBT, cells were stained with DAPI (0.1 µg/mL) in PBT for 10 min and mounted in Vectashield (Vector Labs).

Quantitative IF was performed as described previously (58). Briefly, images were obtained using the Nikon Ti-E inverted microscope with a Zyla sCMOS digital camera with a 40× oil objective. For each biological replicate, all samples were captured at the same magnification and same exposure time. For quantitative analysis of H3K4 methylation levels, regions of interest (ROIs) were defined based on the DAPI signal. The signal intensity of H3K4 methylation levels was extracted for each ROI. The signal was normalized to the DAPI signal intensity to account for differences in the amount of DNA per cell. For each replicate, three images were randomly taken and quantified. Approximately 300 randomly selected cells were used for each biological replicate. Two to three biological replicates were used for data analysis. Kruskal–Wallis one-way ANOVA was performed in GraphPad Prism for statistical significance.

### IP.

IPs were performed as previously described (35). Briefly, 1 g of flash-frozen embryos aged 18 to 24h after egg laying was collected, dechlorinated, and flash-frozen in liquid nitrogen. Frozen embryos were ground with a mortar and pestle in liquid nitrogen and then dissolved in 2 mL of NP40 lysis buffer (50 mM Tris–HCl pH 8.0, 150 mM NaCl, 1% NP40, 1 mM Ethylenediaminetetraacetic Acid (EDTA), and 1 mM Ethylene Glycol-bis(β-aminoethyl Ether)-N,N,N',N'-tetraacetic Acid (EGTA)) with 2× Protease Inhibitor Cocktail (Millipore Sigma cOmplete™) on ice. 30 U/mL of Benzonase was added and incubated on ice for 30 min. The lysate was centrifuged at 4 °C for 5 min at 4,000 relative centrifugal force (RCF), and the supernatant was used for IP with 25 μL of prewashed Pierce™ anti-HA magnetic beads (Sigma-Aldrich). After a 2-h incubation at 4 °C for 2 h, beads were washed three times with NP40 lysis buffer. Proteins were eluted by boiling in 50 μL 2× Laemmli sample buffer supplemented with 50 mM Dithiothreitol (DTT) for 5 min. Three biological replicates were performed for BRWD3-HA embryos and ORR control embryos. The extracts were labeled with TMT for quantitative mass spectrometry as described below.

### TMT Labeling and Tandem Mass Spectrometry.

The IP elutes were verified by western blot (5% of total material), and the remaining material was precipitated using methanol and chloroform. The pellet was washed with methanol to remove excess detergent. Protein was then dissolved in 5 μL fresh 1% RapiGest SF Surfactant (Waters Cat. #186001861) and diluted with 10 μL 0.5 M HEPES (4-(2-hydroxyethyl)-1-piperazineethanesulfonic Acid) pH 8.0 and 32.5 μL H2O. 0.5 μL of a 0.5 M TCEP (Tris(2-carboxyethyl)phosphine) was added to reduce disulfide bonds. One microliter of a 0.5 M iodoacetamide was added to acetylate-free sulfhydryl groups for 30 min at room temperature in the dark. Trypsin digestion was performed overnight at 37 °C with vigorous shaking. Trypsinized samples were labeled with TMT using a TMT10plex kit (Thermo Scientific catalog #90110). The excess label was neutralized with ammonium bicarbonate at a final concentration of 0.4% for 1 h. Samples were mixed and acidified to pH 2 with formic acid. Sample volume was reduced to 1/6th of the original volume using a SpeedVac and restored to the original volume using buffer A (5% acetonitrile and 0.1% formic acid). RapiGest was cleaved by incubating at 42 °C for 1 h. Samples were spun at 14,000 rpm for 30 min, and the supernatant was transferred to a new tube and stored at −80 °C.

### In Vivo Ubiquitination assays.

S2 cells were cotransfected with plasmids expressing FLAG-Ubiquitin and HA-tagged KDM5 for 2 d. For transient transfection in WT and *BRWD3* depleted S2 cells, cells were transfected with 100 ng of FLAG-Ubiquitin-expressing plasmid and 200 ng of HA-KDM5-expressing plasmid using the Effectene II (Invitrogen) kit according to the manufacturer's protocol. For BRWD3 overexpression, cells were cotransfected with 200 ng/mL of BRWD3-V5-expressing plasmid together with 100 ng of a FLAG-Ubiquitin-expressing plasmid and 200 ng of an HA-KDM5-expressing plasmid. To enrich ubiquitinated KDM5 and prevent its degradation, cells were treated with the proteasome inhibitor MG132 for 16 h before harvesting.

To enrich KDM5-HA in denaturing conditions, 2 to 3 million cells were harvested and lysed with 100 μL denaturing buffer (1% Sodium Dodecyl Sulfate, 10 mM Tris–HCl, 150 mM NaCl, 1.0% Triton-X100, 1% sodium deoxycholate, 0.5 mM EDTA, and 10 mM dithiothreitol) supplemented with 2× protease inhibitor and 30 U/mL Benzonase for 20 min on ice and then boiled for 2 min. The lysate was centrifuged at 17,000 × g at 4 °C for 5 min. The supernatant was transferred to a new tube. Then, 900 μL NP40 buffer (50 mM Tris–HCl pH 8.0, 150 mM NaCl, 1% NP40, 1 mM EDTA, and 1 mM EGTA) was added, supplemented with 2× Protease Inhibitor Cocktail. Fifteen microliters of anti-HA beads was added to the lysate and incubated at 4 °C for 2 h. The beads were washed in NP40 buffer three times, and protein was eluted in 50 μL 2× Laemmli sample buffer (with 50 mM DTT) by boiling for 5 min. The elutes were used for subsequent western blotting.

### CHX Chase Assay.

First, RNA interference in S2 cells was performed with 20 µg of double-stranded RNA (dsRNA) targeting *GFP*, *BRWD3*, or *Cul4* for 2 d in six-well plates as described above. Cells were then transfected with 200 μg/mL *HA-KDM5*-expressing plasmid DNA using the Effectene II (Invitrogen) kit according to the manufacturer’s protocol. After 2 d, cells were treated with the translation inhibitor CHX (Sigma) dissolved in dimethyl sulfoxide (DMSO) at 1 mg/mL. Cells were harvested at different time points, centrifuged, and lysed in 50 μL 2× Laemmli sample buffer (with 50 mM DTT) and boiled for 5 min. To inhibit Cullin E3 ubiquitin ligase activity, cells were treated with 50 μM of the Nedd8 inhibitor MLN4924 (Selleckchem). To measure KDM5-HA protein levels, 5 μL of each sample was used for western blot analysis with anti-mouse HA (Cell Signaling, 1:1,000).

### Western Blotting.

Samples were loaded on 4 to 15% Mini-protein TGX (Tris-Glycine eXtended) Satin-free gel (Bio-Rad) for electrophoresis. The gel was activated, imaged, and transferred to a low fluorescence Polyvinylidene Difluoride (PVDF) membrane with a Trans-Blot Turbo Transfer System (Bio-Rad). The blot was imaged directly to measure the total loaded proteins. After blocking in 5% fat-free milk in Tris-Buffered Saline with Tris-HCl (TBST) (140 mM NaCl, 2.5 mM KCl, 50 mM Tris–HCl pH 7.4, and 0.1% Tween 20), blots were incubated with primary antibody for 1 h at room temperature. The primary antibodies used are Mouse anti-HA (Cell Signaling #2367, 1:1,000), Rabbit anti-HA (Abcam ab9110, 1:2,000), Rabbit anti-K48-linkage Specific Polyubiquitin Antibody (Cell Signaling #4289,1:1,000), Rabbit anti-K63-linkage Specific Polyubiquitin Antibody (Cell Signaling #5621,1:1,000), Mouse ANTI-FLAG® M2 antibody (Sigma-Aldrich, 1:1,000), Rabbit anti-BRWD3 (19, 1:500), Rabbit anti-H3K4me3 (Active Motif, 1:1,000), Rabbit anti-H3K4me2 (Cell Signaling, 1:1,000), and Rabbit anti-H3K4me1 (Cell Signaling, 1:1,000). After primary antibody incubation, blots were washed three times in TBST. Blots were then incubated with HRP-conjugated secondary antibody (Jackson Labs, 1:15,000) and/or fluorophore-conjugated secondary antibodies (Bio-Rad, 1:2,000), washed three times, and imaged. To quantify protein levels in western blot, Bio-Rad Image Lab software was used. Band intensities were measured and normalized to the total amount of protein loaded in each lane (based on the total protein on the blot).

### SNAP Spike-in ChIP-seq and qPCR.

H3K4me3 ChIP was performed using ~50 million S2 cells harvested after RNAi treatment and centrifuged at 600 rcf for 5 min. Cells were washed twice with PBS and fixed with 1% PFA for 10 min at room temperature. Cross-linking was quenched by adding glycine to a final concentration of 125 mM and incubating at room temperature for 5 min. Cells were then centrifuged and resuspended in Radioimmunoprecipitation Assay buffer (RIPA) buffer supplemented with 2× complete TM Protease Inhibitor Cocktail EDTA-free (Roche). Chromatin shearing was performed using a Diagenode Bioruptor. To facilitate accurate normalization, 10 µL of sheared chromatin was de-cross-linked overnight and purified, and DNA concentration was measured. One microliter of spike-in SNAP-ChIP K-MetStat mononucleosomes (EpiCypher, Cat. 19-1001) was added to 20 µg of sonicated chromatin. Three micrograms of H3K4me3 antibody (Active Motif, Cat. 39060) was added to the chromatin extract and incubated for 2 h at 4 °C. Prewashed Protein A beads were added to the extract and incubated for 1 h at 4 °C. The beads were then washed twice with RIPA buffer, twice with high-salt RIPA buffer (500 mM NaCl), twice again with RIPA buffer, and finally with TE buffer. The eluted IP sample was divided into two portions. One portion was utilized for constructing a DNA library for next-generation sequencing using the NEBNext Ultra II Library Prep Kit for Illumina (NEB #E7645), following the manufacturer’s protocol. The second portion was used for qPCR analysis.

#### ChIP-qPCR with spike-in normalization.

For the ChIP-qPCR analysis, we used specific primers targeting H3K4me3 peak regions or non-H3K4me3 regions as negative control. Primers targeting the SNAP-ChIP K-MetStat Panel nucleosomes were specified by the manufacturer (EpiCypher). The qPCR reactions were carried out in triplicate using the SYBR Green Master Mix, following the manufacturer's protocol (Bio-Rad, Cat. 1725121). Enrichment values were calculated relative to input samples using the formula: %Enrichment = ((2^CtInput – CtIP^)/percent of Input sample) × 100%. To calculate the spike-in-normalized signal, we used the formula: Normalized signal = (%Input of gene locus)/(% Input of SNAP ChIP). To address the variation in IP efficiency among biological replicates, we normalized all primers to the positive region 1 primer set from the WT sample within each respective replicate. This primer was set as a reference value of 1 for the WT sample in all replicates.

#### ChIP-seq with spike-in normalization.

For each sample, reads were trimmed using fastp to generate trimmed.fastq.gz files. The trimmed reads were then mapped to a custom genome, which included dm6 and concatenated barcode sequences corresponding to the SNAP-ChIP K-MetStat Panel, using bowtie2. The resulting Sequence Alignment/Map (SAM) files were converted to BAM format, sorted, and indexed using samtools. Binary Alignment Map (BAM) files were converted to Browser Extensible Data (BED) format using bedtools bamtobed function. To normalize the data, the total number of reads mapped to the spike-in barcode sequences was counted for each sample. To calculate the scaling factor for each replicate, the summed reads for the spike-in barcodes for the WT samples were divided by the summed reads for the spike-in barcodes of the *BRWD3* knockdown IP sample of the same biological replicate. Finally, the bamCoverage tool was used to generate a bigwig file incorporating the scaling factor with a bin size of 50 bp. Genome coverage tracks were visualized using the Integrative Genomics Viewer-Web (Broad Institute), as in Fig. 1*C*. Heatmaps were generated from spike-in normalized data using previously published H3K4me3 peak regions (generated in ref. 19; GSE101646). Deeptools plotHeatmap was used to generate the mean ChIP-seq signal heatmaps centered on H3K4me3 peaks (dm6) using spike-in normalized bigwig files.

To generate heatmaps of previously published H3K4me3 ChIP-seq fastq files, reads from GSE101646 (19) were aligned to dm3 genome using Bowtie2 with default conditions, and duplicate reads were tagged using with Markduplicates (Broad Institute). Peaks were called with MACS2 callpeak tool with default settings and plots were generated using deepTools plotHeatmap. For BRWD3 and KDM5 ChIP-seq data [GSE101646 (19) and GSE70591 (42)], fastq files were aligned to the dm6 genome using Bowtie2, Markduplicates. MACS2 was used to call peaks. The overlap between BRWD3 and KDM5 peaks was generated using the Intersect intervals tools in bedtools. The *P* value was calculated with a Fisher test between the two peak files using bedtools FisherBed tools.

### Chromatin Marker Enrichment in BRWD3 Peaks.

We downloaded annotations for histone modifications and transcription factor binding sites from modENCODE. The annotations are derived from ChIP-seq and ChIP-chip files in *Drosophila melanogaster* S2 cells (dm3).

We calculated enrichment for overlap between BRWD3 and modENCODE annotations derived from ChIP-seq/ChIP-chip. Overlap was determined by counting the number of basepairs overlapping between the BRWD3 peak and the histone modification or transcription factor binding peak using BEDTools (59). We used a permutation-based strategy to determine whether the number of overlapping base pairs was more or less than expected compared to the null distribution.

We generated the null distribution by randomly shuffling the modENCODE annotation peaks throughout the genome. The random peaks were length-matched with the original set of annotated peaks and exclude any blacklisted or gap regions (60). In addition, for modENCODE data obtained from ChIP-chip, we required that the shuffled peaks maintained the probe density of the original peak in addition to matching on the length. We reshuffled peaks that fell more than 0.1 away from the original probe density until at least 99% of the peaks could be appropriately matched. For each BRWD3 peak and modENCODE annotation, we performed 1,000 permutations. Finally, we calculated an empirical *P* value for the observed amount of overlap by comparing it to the null distribution generated above. We correct the p values for multiple testing using the Benjamini–Hochberg procedure with a false discovery rate < 0.05.

### RNA-seq Analysis.

RNA-seq datasets (GSE101646) were subjected to quality control using FastQC, followed by trimming with Trimmomatic to ensure high-quality data. The cleaned reads were aligned to the Drosophila reference genome (dm6) using the STAR aligner, and the number of reads mapped to each annotated gene was counted using featureCounts. Analysis of differential gene expression was performed using DESeq2, accounting for conditions and replicates. The analysis included two replicates of two control and two BRWD3 dsRNA knockdown samples, resulting in a total of eight samples. Genes with an adjusted p value below 0.05 were deemed differentially expressed. Three BED files were created to represent the gene regions of each gene group: down-regulated, up-regulated, or unchanged. These regions were defined based on the differential gene expression analysis. Then, spike-in normalized H3K4me3 ChIP-seq coverage BigWig files were used to map to H3K4me3 levels in these three regions. Finally, deepTools computeMatrix and plotHeatmap functions were utilized to calculate the coverage matrix and generate a heatmap visualization, respectively. This analysis was performed in Galaxy (usegalaxy.org).

### MudPIT Liquid Chromatography–Tandem Mass Spectrometry.

Triphasic MudPIT columns were prepared as previously described using alternating layers of 1.5 cm C18 resin, 1.5 cm Strong Cation Exchange (SCX) resin, and 1.5 cm C18D (61). Pooled TMT samples (roughly 20 µg of peptide from lysate samples) were loaded onto the microcapillaries using a high-pressure chamber, followed by a 30-min wash in buffer A (95% water, 5% acetonitrile, and 0.1% formic acid). Peptides were fractionated online by liquid chromatography using an Ultimate 3000 nanoLC system and subsequently analyzed using an Exploris480 mass spectrometer (Thermo Fisher). The MudPIT columns were installed on the LC column switching valve and followed by a 20-cm fused silica microcapillary column filled with Aqua C18, 3µm, C18 resin (Phenomenex) ending in a laser-pulled tip. Prior to use, columns were washed in the same way as the MudPIT capillaries. MudPIT runs were carried out by 10 µL sequential injections of 0, 10, 20, 40, 60, 80, and 100% buffer C (500 mM ammonium acetate, 94.9% water, 5% acetonitrile, and 0.1% formic acid), followed by a final injection of 90% C, 10% buffer B (99.9% acetonitrile and 0.1% formic acid v/v). Each injection was followed by a 130-min gradient using a flow rate of 500 nL/min (0 to 6 min: 2% buffer B, 8 min: 5% B, 100 min: 35% B, 105 min: 65% B, 106 to 113 min: 85% B, 113 to 130 min: 2% B). Electrospray Ionization was performed directly from the tip of the microcapillary column using a spray voltage of 2.2 kV, an ion transfer tube temperature of 275 °C, and an RF Lens of 40%. MS1 spectra were collected using a scan range of 400 to 1,600 m/z, 120 k resolution, AGC target of 300%, and automatic maximum injection times. Data-dependent MS2 spectra were obtained using a monoisotopic peak selection mode: peptide, including charge state 2 to 7, TopSpeed method (3 s cycle time), isolation window 0.4 m/z, HCD fragmentation using a normalized collision energy of 32%, resolution 45 k, AGC target of 200%, 120 ms (IP) maximum injection times, and a dynamic exclusion (20 ppm window) set to 60 s.

### Peptide Identification and Quantification.

Identification and quantification of peptides were performed in Proteome Discoverer 2.4 (Thermo Fisher) using a UniProt *D. melanogaster* proteome database (downloaded February 6th, 2019) containing 21,114 protein entries. The database was adjusted to remove splice-isoforms and redundant proteins and supplemented with common MS contaminants. Analyses were conducted with Sequest HT using the following parameters: trypsin cleavage (maximum 2 missed cleavages), minimum peptide length 6 AAs, precursor mass tolerance 20 ppm, fragment mass tolerance 0.02 Da, dynamic modifications of Met oxidation (+15.995 Da), protein N-terminal Met loss (−131.040 Da), and protein N-terminal acetylation (+42.011 Da), static modifications of TMT 10plex (+229.163 Da) at Lys and N termini and Cys carbamidomethylation (+57.021 Da). Peptide IDs were filtered using a percolator with an false discovery rate (FDR) target of 0.01. Proteins were filtered based on a 0.01 FDR, and protein groups were created according to a strict parsimony principle. TMT reporter ions were quantified considering unique and razor peptides, excluding peptides with coisolation interference greater than 25%. Peptide abundances were first normalized based on total peptide amounts in each channel, assuming similar levels of background in the IPs. The abundance-normalized peptide amounts were then normalized to medium peptide amount in each channel. The Student *t* test was performed between ORR-IP groups and HA-IP groups (*P* < 0.05).

## Supplementary Material

Appendix 01 (PDF)

Dataset S01 (XLSX)

Dataset S02 (XLSX)

Dataset S03 (XLSX)

## Data Availability

The mass spectrometry proteomics data have been deposited to the ProteomeXchange Consortium via the PRIDE (62) partner repository with the dataset identifier PXD039708. Genomic datasets generated in this study have been deposited in the GEO database under accession number GSE236629. Previously published data were used for this work (19) and GSE101646.
