# Supplementary material for: BRWD3 promotes KDM5 degradation to maintain H3K4 methylation levels
Source: Proc Natl Acad Sci U S A. 2023 Sep 18;120(39):e2305092120. doi: 10.1073/pnas.2305092120 (PMC10523488; doi:10.1073/pnas.2305092120)
Supplement: Supplementary file 1 — Appendix 01 (PDF) [file pnas.2305092120.sapp.pdf]

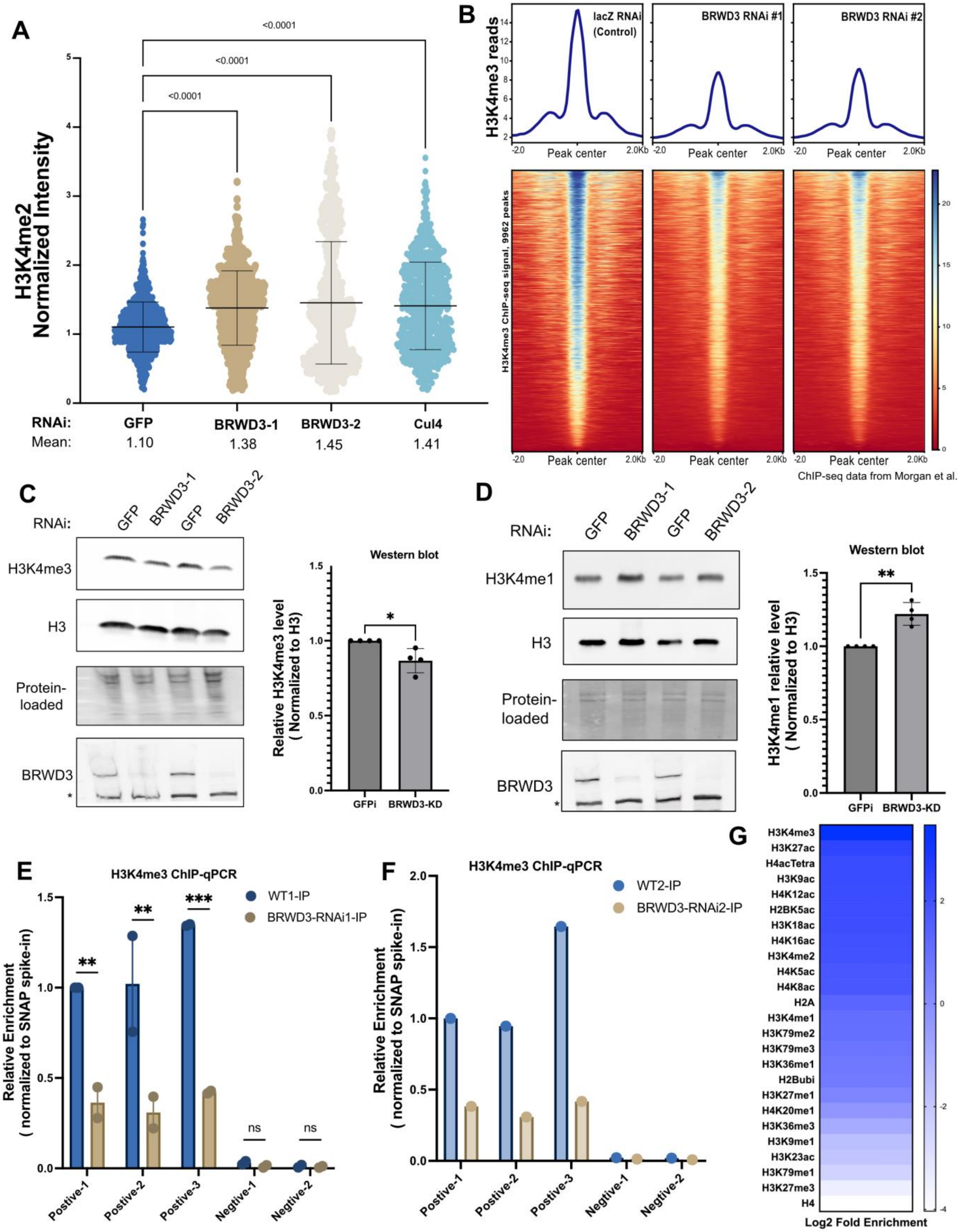
A
H3K4me2 Normalized Intensity
<0.0001
<0.0001
<0.0001
RNAi: GFP BRWD3-1 BRWD3-2 Cul4
Mean: 1.10 1.38 1.45 1.41
B
H3K4me3 reads
lacZ RNAi (Control)
BRWD3 RNAi #1
BRWD3 RNAi #2
Peak center
H3K4me3 ChIP-seq signal, 9962 peaks
ChIP-seq data from Morgan et al.
C
RNAi: GFP BRWD3-1 GFP BRWD3-2
H3K4me3
H3
Protein-loaded
BRWD3
Western blot
Relative H3K4me3 level (Normalized to H3)
GFPi BRWD3-KD
D
RNAi: GFP BRWD3-1 GFP BRWD3-2
H3K4me1
H3
Protein-loaded
BRWD3
Western blot
H3K4me1 relative level (Normalized to H3)
GFPi BRWD3-KD
E
H3K4me3 ChIP-qPCR
WT1-IP
BRWD3-RNAi1-IP
Relative Enrichment (normalized to SNAP spike-in)
Postive-1 Postive-2 Postive-3 Negtive-1 Negtive-2
F
H3K4me3 ChIP-qPCR
WT2-IP
BRWD3-RNAi2-IP
Relative Enrichment (normalized to SNAP spike-in)
Postive-1 Postive-2 Postive-3 Negtive-1 Negtive-2
G
H3K4me3
H3K27ac
H4acTetra
H3K9ac
H4K12ac
H2BK5ac
H3K18ac
H4K16ac
H3K4me2
H4K5ac
H4K8ac
H2A
H3K4me1
H3K79me2
H3K79me3
H3K36me1
H2Bubi
H3K27me1
H4K20me1
H3K36me3
H3K9me1
H3K23ac
H3K79me1
H3K27me3
H4
Log2 Fold Enrichment

Supplemental Figure 1. BRWD3 affects H3K4 methylation status. (A) Violin plots of quantitative immunofluorescence experiments in Drosophila S2 cells using an anti-H3K4me2 antibodies. Each distribution represents the signal intensities of 1000 randomly selected cells from three biological replicates. ****p < 0.0001 ANOVA one-way analysis with Tukey's multiple comparisons test. (B) Enrichment heatmap of H3K4me3 ChIP-seq signal sorted by mean occupancy around the centers of all H3K4me3 peaks. (C) Anti-H3K4me3 Western blot analysis of BRWD*3* depleted S2 cells with anti-H3 and total protein loading controls. Anti-BRWD3 blot shows the knockdown efficiency. The quantification and normalized to total H3 levels are shown on the right. Four replicates, unpaired t-test (*p < 0.05). (D) Similar experiments as in (C), but with an anti-H3K4me1 antibody. Four replicates, unpaired t-test (**p < 0.01). (E) Spike-in normalized H3K4me3 ChIP-qPCR with no dsRNA treatment control or *BRWD3* dsRNA1 knockdown. Data points represent two biological replicates. Relative enrichment to positive primer set 1 was measured. Positive primers correspond to known H3K4me3 regions while negative primers target non-H3K4me3 regions. Two-way ANOVA with Šídák's multiple comparisons test (**p < 0.01, ***p < 0.001). (F) similar experiments as in (E) but with *BRWD3* dsRNA2 knockdown. (G) Significant enrichment of all published histone modifications within BRWD3 binding sites. Log2-fold enrichment for observed overlap relative to expected overlap for each mark peak set is shown.

A

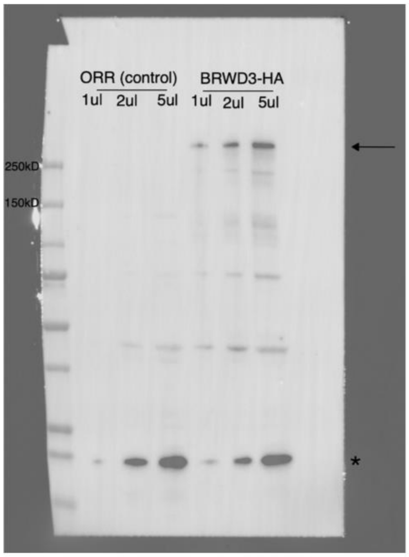

Supplemental Figure 2. Verification of endogenously HA-tagged BRWD3. Western blot samples were prepared from endogenously HA-tagged BRWD3 embryos (0-24h) or ORR negative control embryos (0-24h), probing with an anti-HA antibody. Arrow indicates BRW3-HA (~270 kD). Asterisk indicates non-specific bands recognized by anti-HA antibody (both in ORR control and BRWD3-HA).

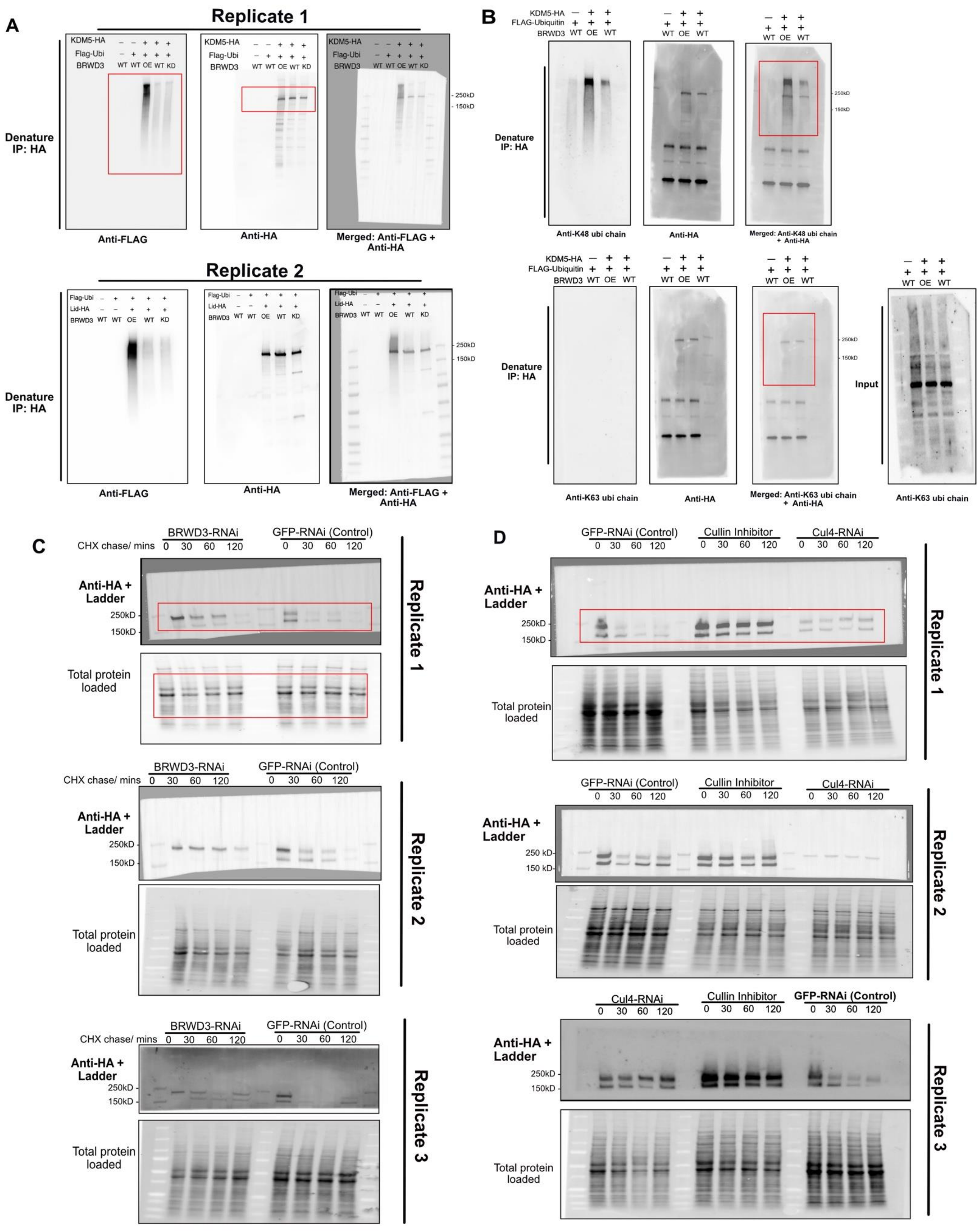
A
Replicate 1
KDM5-HA
Flag-Ubi
BRWD3
WT WT OE WT KD
Denature
IP: HA
250kD
150kD
Anti-FLAG
Anti-HA
Merged: Anti-FLAG + Anti-HA
Replicate 2
Flag-Ubi
Lid-HA
BRWD3
Anti-FLAG
Anti-HA
Merged: Anti-FLAG + Anti-HA
B
KDM5-HA
FLAG-Ubiquitin
BRWD3
WT OE WT
Anti-K48 ubi chain
Anti-HA
Merged: Anti-K48 ubi chain + Anti-HA
Anti-K63 ubi chain
Anti-HA
Merged: Anti-K63 ubi chain + Anti-HA
Input
Anti-K63 ubi chain
C
BRWD3-RNAi
GFP-RNAi (Control)
CHX chase/ mins
0 30 60 120
Anti-HA + Ladder
Total protein loaded
Replicate 1
Replicate 2
Replicate 3
D
GFP-RNAi (Control)
Cullin Inhibitor
Cul4-RNAi
0 30 60 120
Anti-HA + Ladder
Total protein loaded
Replicate 1
Replicate 2
Replicate 3

Supplemental Figure 3. BRWD3 promotes KDM5 ubiquitination and degradation. Full Western blots and additional replicates used for Figure 3 in the main text. Red boxes indicate cropped regions. (A) Two biological replicates for the denaturing HA-immunoprecipitation followed by Western blots in Fig. 3A are shown. Blots were probed with anti-FLAG (left) and anti-HA (middle) antibodies. The transfection conditions and *BRWD3* expression conditions are indicated on the top. (B) Full Western blots for ubiquitin chain-type specific antibody probing after denaturing HA-IP in Fig. 3B. Separated channels for anti-K48-linked polyubiquitin chains (upper, left) and anti-HA (upper, middle) antibodies, and merged channels (upper, right). Similar order is shown with an anti-K63-linked polyubiquitin antibody (lower). The input samples were blotted with anti-K63-linked polyubiquitin antibody and showed signal (lower, right). (C) Full Western blots for cycloheximide assays under *BRWD3*-RNAi or *GFP*-RNAi conditions used in Fig. 3D. Two more replicates are shown below. (D) Full Western blot for cycloheximide assays under *Cul4*-RNAi, Cullin inhibitor incubation, or *GFP*-RNAi conditions used in Fig. 3F.

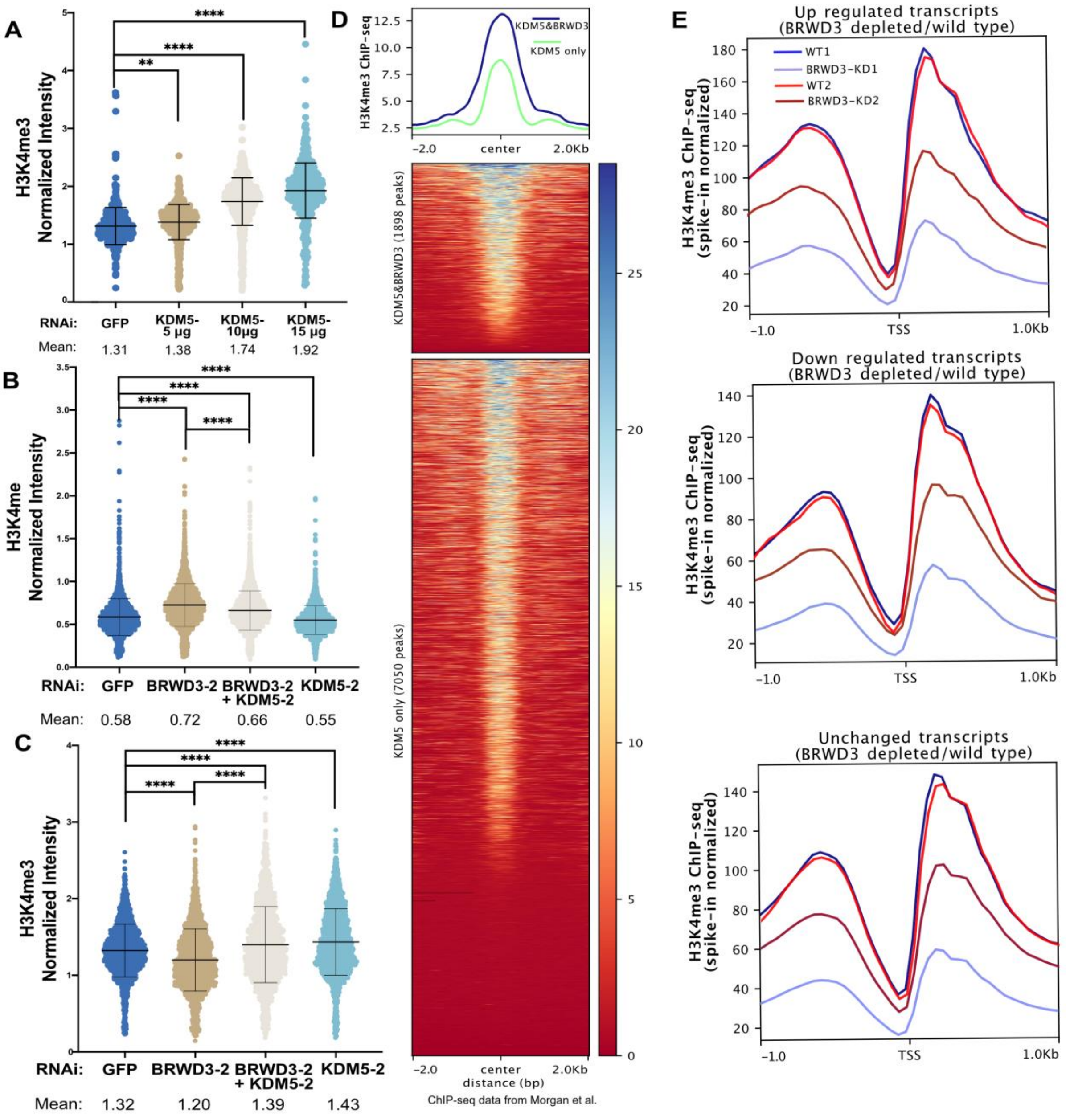

Supplemental Figure 4. Inhibition of KDM5 with an independent set of dsRNAs restores altered H3K4 methylation levels upon BRWD3 depletion. Violin plot quantitative immunofluorescence assays plot in Drosophila S2 cells using an anti-H3K4me3 antibody (A, B) or an anti-H3K4me1 antibody (C) with different dsRNA treatments. **p< 0.01, ****p < 0.0001 using ANOVA one-way analysis with Tukey's multiple comparisons test. (D) Heatmap illustrating the enrichment of H3K4me3 ChIP-seq signal, organized by the average occupancy around the centers of two distinct groups of peaks: sites occupied by BRWD3 and KDM5, or sites solely associated with KDM5. Published ChIP-seq Data (dm3) from Morgan et al. was used. (E) Quantification of spike-in normalized H3K4me3 ChIP-seq signal (from Fig. 1D) around three distinct groups of gene regions based on differential gene expression profile (see Table S3). The analysis was conducted using two replicates and two different BRWD3 dsRNAs (GSE101646).
